# Facial Expression Recognition Using Local Sliding Window Attention

**DOI:** 10.3390/s23073424

**Published:** 2023-03-24

**Authors:** Shuang Qiu, Guangzhe Zhao, Xiao Li, Xueping Wang

**Affiliations:** 1 School of Electrical and Information Engineering, Beijing University of Civil Engineering and Architecture, Beijing 100044, China; 2 Beijing Key Laboratory of Robot Bionics and Function Research, Beijing 100044, China; 3 School of Electronics and Information Engineering, Zhongyuan University of Technology, Zhengzhou 450007, China

**Keywords:** facial expression recognition, sliding window, local feature enhancement, adaptive feature selection

## Abstract

There are problems associated with facial expression recognition (FER), such as facial occlusion and head pose variations. These two problems lead to incomplete facial information in images, making feature extraction extremely difficult. Most current methods use prior knowledge or fixed-size patches to perform local cropping, thereby enhancing the ability to acquire fine-grained features. However, the former requires extra data processing work and is prone to errors; the latter destroys the integrity of local features. In this paper, we propose a local Sliding Window Attention Network (SWA-Net) for FER. Specifically, we propose a sliding window strategy for feature-level cropping, which preserves the integrity of local features and does not require complex preprocessing. Moreover, the local feature enhancement module mines fine-grained features with intraclass semantics through a multiscale depth network. The adaptive local feature selection module is introduced to prompt the model to find more essential local features. Extensive experiments demonstrate that our SWA-Net model achieves a comparable performance to that of state-of-the-art methods with scores of 90.03% on RAF-DB, 89.22% on FERPlus, 63.97% on AffectNet.

## 1. Introduction

Facial expressions are the most intuitive and effective body language symbols to convey emotions. Facial expression recognition (FER) enables machines to recognize human facial expressions automatically. FER has made a massive contribution to the fields of human–computer interactions [1], vehicle-assisted driving [2], medical services [3], and social robots [4]. FER is becoming an increasingly active field, and great progress has been made in this area in the past few decades.

Excellent recognition results [5,6] have been achieved on datasets collected in controlled laboratory environments, such as CK+ [7], MMI [8], and JAFFE [9]. However, in the wild, FER is still far from satisfactory. Occlusions and head pose variations are the common, intractable problems experienced in unconstrained settings. Therefore, researchers have proposed diverse real-world facial expression datasets such as FER2013 [10], RAF-DB [11], AffectNet [12] to advance the development of FER in the wild. The authors of [13,14,15,16] have made great contributions based on datasets of natural scenes. Li et al. [15] used the face as a whole to obtain various global features to produce a competitive performance, but they ignored the importance of local features. Occlusion or the appearance of poses in the wild can cause the effective information about the face to be incomplete. Therefore, it is difficult to mine significant expression features, resulting in the model having a poor discrimination ability. Karnati et al. [5] and Ruan et al. [6] showed that the learning of fine-grained features plays a huge role in expression recognition, thus considering both global features and local features is a better choice.

**Figure 1:**
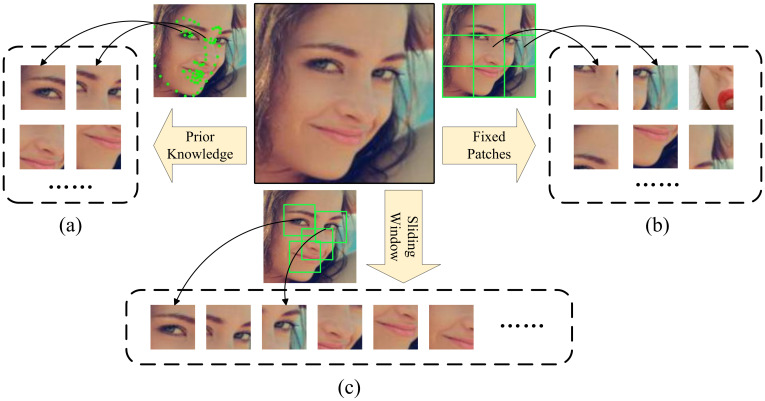
Different cropping strategies for facial expression images. (**a**) Cropping based on prior knowledge. Green dots represent facial landmarks. Positioning and cropping can be performed according to point coordinates. (**b**) Cropping based on fixed-size patches. The face image is equally divided into 9 regions. (**c**) Cropping based on sliding window. The window scans the entire face image and accurately locates the key regions of the face.

Most current methods for obtaining local features crop essential areas of the face through prior knowledge [16,17,18] or directly crop the face into patches of fixed size [14,19]. However, these methods have some problems: (1) The method of cropping faces through prior knowledge is in the data preprocessing stage, and most research methods conduct cropping based on landmarks. Although this method is intuitive, facial keypoint detection is prone to the effects of occlusion and pose. As shown in Figure 1a, landmarks are not accurately located. Therefore, the cropping method based on prior knowledge itself contains certain noise. (2) The cropping of images into fixed-scale patches does not require prior knowledge but may divide useful features into different patches. As shown in Figure 1b, eyes are an essential feature for judging expressions, and direct segmentation may divide the same eye into several different patches, causing the integrity of essential features to be destroyed. Thus, the sliding window-based cropping strategy is introduced to obtain the complete feature, as shown in Figure 1c. It requires no prior knowledge and ensures that essential features are not segmented, which is beneficial for FER.

Some studies [14,16,19] have obtained multiple local features and directly used them for FER; however, some useless features are learned because some local features contain background and noise information, which is not conducive to FER. Therefore, we enhance the local features to obtain multiscale fine-grained feature information after obtaining multiple local features. In addition, we further propose a local feature fusion method to select and fuse the enhanced local features. By assigning different weights, reliable features are given greater weights, and useless features are given smaller weights. This ensures that our method can adaptively identify more useful local features from different parts.

In summary, we propose a local Sliding Window Attention Network (SWA-Net) to mitigate the occlusion and variable pose effects of FER. Firstly, the backbone network is used to directly obtain global feature information with a global receptive field. Moreover, we utilize the Sliding Window Cropping (SWC) strategy to crop the coarse-grained features from the global branch output to obtain a set of subfeature blocks containing complete local information. Additionally, the Local Feature Enhancement (LFE) module is employed to obtain local multiscale fine-grained features. Finally, we introduce the Adaptive Feature Selection (AFS) module to focus on more important local features.

In brief, the contributions of our study are summarized as follows.

- We propose a Sliding Window Cropping strategy that can crop out all local regions to alleviate the negative effects of occlusion and pose experienced in the wild. This method neither requires complex preprocessing nor destroys the integrity of the features.
- We propose the Local Feature Enhancement module to extract more fine-grained features containing semantic information.
- The Adaptive Feature Selection module is introduced to help the model to autonomously find discriminant features from the local information.
- We conduct comprehensive experiments on widely used wild datasets and specialized occlusion and pose datasets to certify the superiority of the SWA-Net method. Our method shows the ability to solve occlusion and pose problems in the wild.

## 2. Related Work

Our method jointly considers global and local information for FER in the wild. Thus, we mainly review in-the-wild FER methods and local feature-based FER methods.

### 2.1. Facial Expression Recognition in the Wild

Some traditional methods use feature engineering, including SIFT [20], HOG [21], and Gabor [22], to focus on global facial information. This has achieved good results. Convolutional Neural Networks (CNN) have been employed with success in the image field, and Fasel et al. [23] found that they have strong robustness to facial pose and scale in face recognition tasks. Tang et al. [24] and Kahou et al. [25] designed deep CNN models to win the championships of FER2013 and Emotiw2013. With the development of GPU hardware, more and more models using deep CNN are being developed. FER based on deep learning has gradually gained the upper hand and become a mainstream research method. Researchers rely on powerful deep learning to quickly overcome the challenge associated with FER in a controlled environment.

More and more researchers are turning their attention to challenging wild environment conditions and working on solving facial recognition problems under natural conditions [26] including illumination changes, occlusions, head pose variations, and blur. Karnati et al. [5] proposed the use of multiscale convolution to obtain more fine-grained features and reduce intraclass differences, aiming to solve the illumination problem. Zhang et al. [27] proposed the use of “uncertainty” learning to quantify the degree of “uncertainty” for various noise problems in facial expressions, mixing “uncertainty” features from different faces to separate noise and expression features. Zou et al. [28] regarded expressions as a weighted sum of different types of expressions and learned basic expression features through a sequential decomposition mechanism. Fan et al. [29] designed a two-stage training program to further recognize expressions using identity information. Zhang et al. [13] proposed Erasing Attention Consistency, which mines the features of the expression itself, rather than the label-related features, to suppress the learning of noise information during training. Ruan et al. [6] learned intraclass features and interclass features by decomposing and reconstructing. Jiang et al. [30] proposed an identity and pose disentangled method, which separates expression features from the identity and pose.

### 2.2. Facial Expression Recognition Based on Local Features

Due to the enormous difficulty in recognizing expression images in the natural environment, guiding models to mine local significant features has become the choice of more researchers. Local-based FER uses some strategies to crop the face image into several local regions and solves the noise problem associated with facial images in the real world by obtaining local information. Li et al. [18] cut out 24 small patches according to the landmark coordinates, generated corresponding weights according to the degree of occlusion, and then predicted the result with the global features. Wang et al. [17] considered multiple cropping strategies and proposed the RB-Loss method to assign different weights to different regions. Zhao et al. [19] reduced the occlusion and pose interference through the use of multiscale features and local attention modules. Liu et al. [16] proposed adaptive local cropping, and particularly cropped the eye and mouth parts, guiding the model to find more distinguishable parts. This method is robust to occlusion and pose changes. Krithika et al. [31] segmented the face and background information and then cut out the eyes, nose, and mouth and proposed a Minimal Angular Feature-Oriented Network to obtain specific expression features. Xue et al. [14] guided the model to learn diverse information within patches and identified rich relationships between patches. Although these methods focus on local features through facial key point cropping or fixed-size cropping, the former will cause inaccurate key point positioning due to facial occlusion and head poses, and the latter may divide essential features into different patches, thereby destroying the integrity of features. Even if this effect is mitigated by mixing the two cropping strategies, the model will still ignore finer expression details, such as brows and muscle lines.

In order to completely crop all local regions used for modeling autonomous mining features, we propose a cropping strategy based on sliding windows. We crop global coarse-grained features using feature-level operations to obtain a set of fine-grained local features that includes important regions. Then, we generate different weights according to the contribution of each subfeature block to the prediction result. Finally, the result is jointly predicted using both global and local information.

## 3. Proposed Method

**Figure 2:**
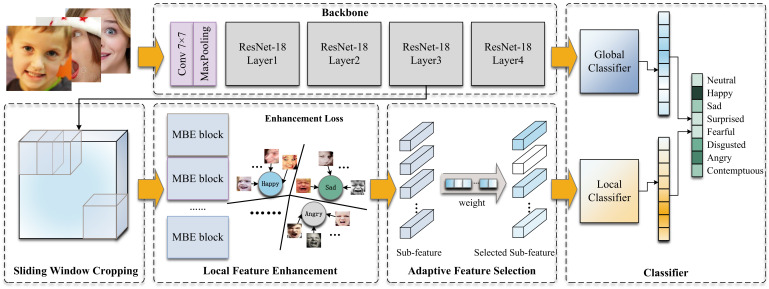
Overview of the SWA-Net model. ResNet-18 serves as the backbone to mine global features. Local features are obtained through the Sliding Window Cropping module, Local Feature Enhancement module, and Adaptive Feature Selection module. Finally, the global classifier and local classifier are used independently to jointly predict the results.

Figure 2 shows an overview of our SWA-Net model. Firstly, we extract the global features with the largest receptive field by the backbone. The local branch consists of the Sliding Window Cropping module, the Local Feature Enhancement module, and the Adaptive Feature Selection module, which are used to supervise our model to mine more distinguishing local features.

We use ResNet-18 [32] as the backbone, which removes the last AvgPooling and FC. In the SWC module, we take the coarse-grained feature from the global branch output as the input and use the sliding window strategy to perform feature-level cropping to obtain a set of local features. The LFE module extracts rich fine-grained feature information through the convolution of different scales and depths of the MultiBranch Enhancement (MBE) block and uses the enhancement loss suppression model to learn noise information in local feature blocks. The AFS module assigns a weight to each enhanced local feature block to guide our model to find more distinguishing local regions spontaneously. Finally, we predict the result with the global and local features jointly.

### 3.1. Sliding Window Cropping Module

**Figure 3:**
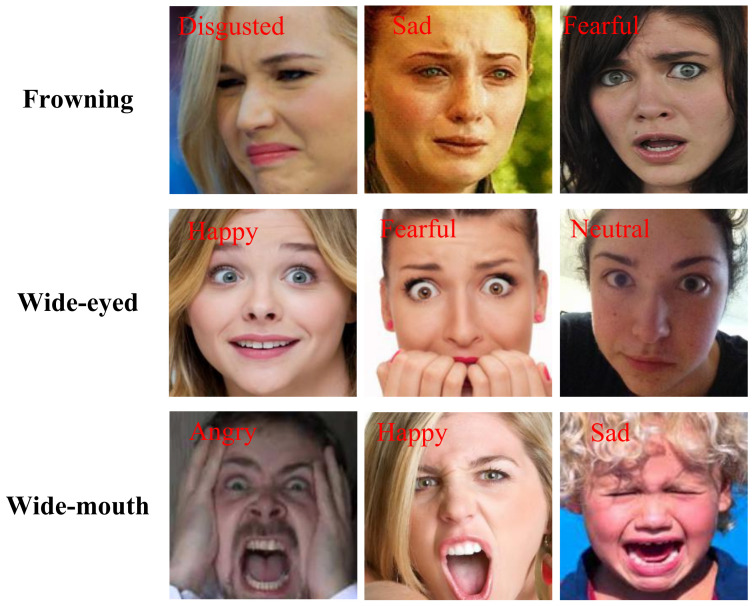
Different expressions have the same facial movements. The black font on the left indicates the similar features in the images. The upper left red font in each face image indicates the expression category of the image.

The effective area of face images is incomplete due to facial occlusion and head poses, so we need to observe different regions of the face at the same time, just as humans usually need to consider multiple areas of the face comprehensively when judging facial expressions. As shown in Figure 3, different expressions have similar actions. For example, in the first row, the three expressions of disgusted, sad, and fearful all have the action of “frowning”. If we only focus on the eyebrows, we cannot accurately identify the expression. However, if we integrate other areas of the face, the eyes of the person in the disgusted image are slightly squinted, and the mouth is closed; the eyes of the person in the sad image may contain tears, and the mouth and nose are tight; and in the fearful image, both the eyes and mouth are wide open. Thus, we can accurately identify the expression from each face image. The SWC module aims to accurately cut out all the important features while ensuring the integrity of the local features through the sliding window cropping strategy.

**Figure 4:**
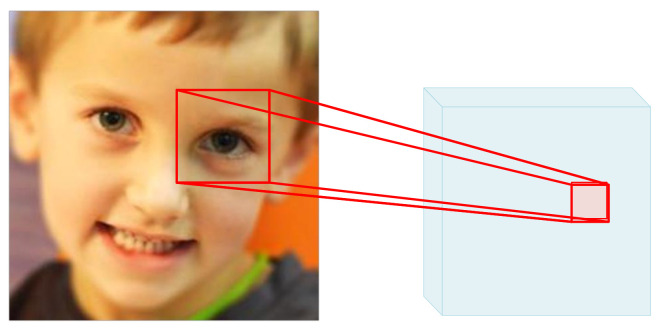
Mapping of the feature map and the raw image. On the coarse-grained feature map, each pixel contains 16×16 pixels of feature information in the raw image. Therefore, it is more efficient to crop the feature map, and it is easier to extract important overall feature information.

In existing works, RAN [17] and AMP-Net [16] use image-level cropping, but the sizes and positions of the eyes from different facial images in the dataset are very different, so it was difficult for us to choose a suitable local region size to cover all of the important features. In this study, we adopted a feature-level cropping strategy. We cropped the coarse-grained feature, covering as many of the important features as possible, because the information of each pixel already included a small range of overall information from the original image, as shown in Figure 4. For example, the input image was 3×224×224, and the feature map after feature pre-extraction was 256×14×14, so the information on each position already included the overall information for 16×16 pixels in the original image.

This alleviates the problem of inappropriate local feature cropping caused by the values of the scale and stride to a certain extent, and it can greatly reduce the amount of calculation required without losing feature information. $O_{i}$ represents the coarse-grained features extracted by the backbone network for the *i*-th image in a mini-batch. $x_{i}=\left\{x_{i}^{1},x_{i}^{2},...,x_{i}^{n}\right\}$ denotes a set of local subfeatures after coarse-grained feature cropping, and $x_{i}^{n}$ refers to the *n*-th local subfeature of the *i*-th image. *Y* represents the set of the corresponding label. Mathematically,

(1) $$O_{i}=F_{pre}\left(X_{i}\right),$$

where $X_{i}$ refers to the *i*-th image, and $F_{pre}$ represents the feature pre-extraction network (the first three layers of the backbone network). Then, the cropping operation can be formulated as

(2) $$x_{i}=\left\{x_{i}^{1},x_{i}^{2},\ldots ,x_{i}^{n}\right\}=SWC\left(O_{i}\right).$$

The value of *n* is related to the scale and stride. In order to balance the accuracy, running time, and computing power, we do not consider the case of stride=1. The specific calculation is as follows:

(3) $$n=[\frac{s_{O}-\left(scale-1\right)}{stride}{]}^{2},$$

where $s_{O}$ refers to the size of the feature map. For example, in this experiment, we set the parameters to scale=7 and stride=2, and we obtained 16 subfeature blocks of 256×7×7 pixels.

### 3.2. Local Feature Enhancement Module

**Figure 5:**
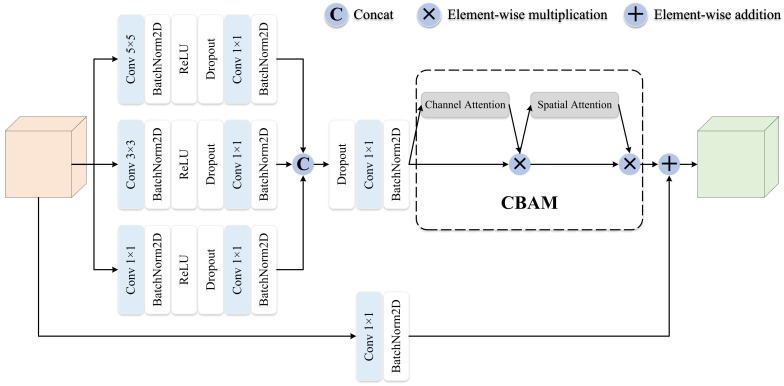
The framework of the MBE block. The MBE consists of a set of multiscale convolution and attention modules.

The LFE module utilizes the MBE block for local features to mine multiscale and deeper feature information to allow a better FER performance. As shown in Figure 5, the MBE block framework consists of three different sizes of convolutions, which are used to mine multiscale features under different receptive fields, and then further learning of deep feature information is carried out by the 1×1 convolution adjustment dimension.

After concatenating the multiscale features, channel enhancement and spatial enhancement are performed through CBAM [33]. Finally, the residual structure is utilized and the feature is added to the enhanced multiscale depth feature, reducing the original feature dimension by a 1×1 convolution. Because of the residual structure, the results produced at this time emphasize the fine-grained feature information learned jointly under different receptive fields without losing the original features. Mathematically,

(4) $$F_{MS}\left(x_{i}\right)=C_{1\times 1}\left(cat\left(F_{1\times 1}\left(x_{i}\right),F_{3\times 3}\left(x_{i}\right),F_{5\times 5}\left(x_{i}\right)\right)\right),$$

(5) $$e_{i}=C_{1\times 1}\left(x_{i}\right)⊕CBAM\left(F_{MS}\left(x_{i}\right)\right),$$

where $C_{1\times 1}$ means matching dimensions through an 1×1 convolution. $F_{MS}$ represents a set of multiscale convolution operations and an 1×1 dimensionality reduction convolution. F is a set of convolution operations, and the subscript is the size of the convolution kernel. $e_{i}$ represents the *i*-th enhanced local subfeature. ⊕ stands for element-wise addition.

Since local information is greatly affected by image noise, the strong learning ability of the enhancement module may lead the model to learn noise information from local feature blocks. Therefore, we designed an enhancement loss based on the idea of CenterLoss [34] to suppress the learning of noise information and enable each subfeature block to learn features with intraclass semantics. Thus, mathematically we can formulate the loss function as

(6) $$L_{enhance}=\frac{1}{n}\cdot \sum_{i=1}^{N}\sum_{j=1}^{n}{∥e_{i}^{j}-c_{i}∥}_{2}^{2},$$

where *n* represents the number of subfeatures. *N* represents the number of images in a mini-batch. ${∥\cdot ∥}_{2}$ represents the L2 norm, and $c_{i}$ stands for the center of the *i*-th image.

### 3.3. Adaptive Feature Selection Module

**Figure 6:**
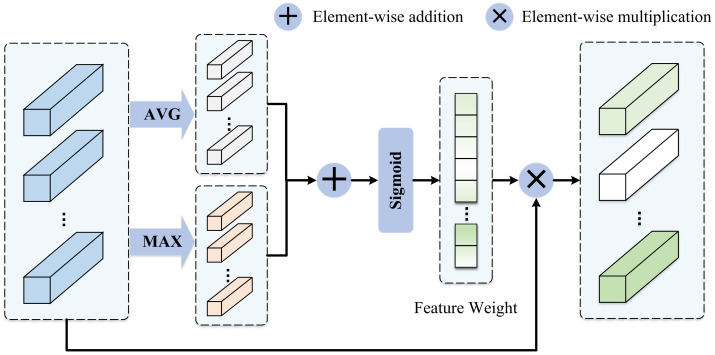
The framework of the AFS block. The AFS block adaptively selects more important local subfeatures by generating a set of feature weights.

Not all areas of the face will be helpful for expression recognition. Emery et al. [35] and Martinez et al. [36] explained the relationship between different expressions and local facial actions. Similarly, not all subfeature blocks obtained by cropping are useful. In other words, all subfeature blocks contribute differently to the prediction result, so we need to select the most discriminant local feature blocks and suppress the influence of useless subfeature blocks or even noise feature regions before predicting the result. As shown in Figure 6, we propose the AFS block in order to make the model find important local features autonomously.

Firstly, for all sub-blocks, the average value and maximum value of the features in the block are respectively calculated and added. Secondly, the sigmoid activation function is used to generate a corresponding weight according to the feature value of each sub-block. Finally, the original subfeature blocks are given different weights, and the important features are enhanced due to having greater weights, and the useless features are weakened. At this time, the model has learned to adaptively select more useful local features according to their contribution degrees. Specifically, this is expressed as

(7) $${\mathrm{x\_selected}}_{i}=\sigma \left(Pooling_{avg}\left(e_{i}\right)+Pooling_{max}\left(e_{i}\right)\right)⊗e_{i},$$

where σ stands for the sigmoid activation function. $Pooling_{avg}$ and $Pooling_{max}$ represent the average pooling and maximum pooling, respectively. ⊗ stands for element-wise multiplication.

### 3.4. Fusion Strategy

Our experiments consider both global and local features to mine facial expression features more comprehensively. For global features, we obtain 512×7×7 features through the backbone network and then obtain the prediction results through a global classifier (GC) containing two linear layers, a batchnorm1d layer and a dropout layer; for local features, we use a GC-like method for each subfeature to obtain *n* one-dimensional features with the length of 64 and then use a linear layer to obtain the prediction results after concatenation. We use a decision-level fusion strategy and set a hyperparameter α to obtain the weights of global and local predictions. Mathematically,

(8) $$\hat{Y}=\alpha \cdot {\hat{Y}}_{global}+\left(1-\alpha \right)\cdot {\hat{Y}}_{local},$$

where $Y_{global}$ stands for the prediction of the global feature. $Y_{local}$ stands for the prediction of the local feature.

In this study, Cross-Entropy Loss was used to measure the prediction results of the global and local features. We set another hyperparameter β as the coefficient of enhancement loss. According to the above analysis, we obtained the final loss function:

(9) $$L=\alpha \cdot L_{global}+\left(1-\alpha \right)\cdot L_{local}+\beta \cdot L_{enhance}.$$

## 4. Experimental Results

### 4.1. Datasets & Evaluation Criteria

#### 4.1.1. Datasets

We performed comprehensive experiments on three widely used facial expression datasets to show the superiority of the SWA-Net model. To certify that our method strongly generalizes facial occlusions and head poses, we also conducted experiments on datasets containing facial occlusion and various head poses [17].

**RAF-DB**: RAF-DB [11] contains about 30,000 face images with multiple occlusions and different head poses which are carefully labeled with seven labels (6 basic expressions and neutral expression). These face images are very challenging to recognize due to the noise factor in the wild. We selected 12,271 and 3068 single-label images from the dataset for training and testing, respectively.

**FERPlus**: FERPlus [37] is based on FER2013 [10]. Images are relabeled into 8 expression categories (6 basic expressions, neutral expression, and contemptuous expression) by 10 crowdsourced labelers, providing higher quality labels than the original FER dataset. However, all of the 35,000 images are cropped with a 48×48 human face. The whole dataset is divided into a training set, validation set, and test set, and we only validated our results on the test set.

**AffectNet**: AffectNet [12] is a very-large-scale dataset of real-world facial expressions containing the largest number of images, more than one million, to date. About 284,000 images are manually labeled. We used 7 expressions (as RAF-DB) for our study. Since AffectNet is a dataset with an extremely unbalanced class distribution, we used an undersampling method with only 60,000 and 3500 images employed for training and testing.

**Occlusion & Pose datasets**: Wang et al. [17] collected these datasets, which contain a wide variety of facial occlusions and multiangle head poses. The occlusion datasets (Occlusion-RAF-DB, Occlusion-FERPlus, Occlusion-AffectNet) contain facial occlusion images including 735, 605, and 592 images, respectively. The pose datasets (Pose30-RAF-DB, Pose30-FERPlus, Pose30-AffectNet, Pose45-RAF-DB, Pose45-FERPlus, and Pose45-AffectNet) contain images of facial pose changes. There are 1247, 1170, and 1749 images with head poses of greater than 30 degrees, and 558, 633, and 889 images with head poses of greater than 45 degrees.

#### 4.1.2. Evaluation Criteria

For a fair comparison with state-of-the-art works, we chose the widely used overall accuracy (Acc.) parameter as the evaluation criteria for all datasets. Specifically, we counted the total number of correct predictions from all expression categories and the total number of expression samples, and then we calculated the percentage of correct predictions, where a higher percentage indicated a better model performance.

### 4.2. Implementation Details

We resized all images to 224×224 pixels and used random horizontal flipping and RandomErasing to avoid overfitting. The experimental setup was the same as that used in RUL [27]. The backbone was ResNet-18 pre-trained on Ms-Celeb-1M [38]. By default, we set the cropping parameters to scale=7 and stride=2 and the hyperparameters to α=0.5 and β=0.0001. Our work was performed with the PyTorch toolbox on the GeForce RTX 2080 Ti platform. For all experiments, we used the Adam optimizer with a weight decay of 0.0001. The learning rate was initialized to 0.0002 and was adjusted by the ExponentialLR with a gamma value of 0.9. The training process lasted for 60 epochs, and each batch size was 64.

### 4.3. Ablation Study

We performed a refined ablation analysis on the RAF-DB dataset to demonstrate the effectiveness of the SWA-Net model. In the experiment, we analyzed each module of the SWA-Net, the scale and stride parameters, and the hyperparameters α, β of the fusion strategy.

In the experiment, we conducted a series of ablation analyses for three modules of the SWA-Net. We chose ResNet-18 [32] as the baseline network. Specifically, we discarded the last AvgPooling layer and FC layer and instead used a 512×7×7→7 FC layer to directly predict the result. On this basis, we added the Sliding Window Cropping module, the Local Feature Enhancement module, and the Adaptive Feature Selection module, in turn, and calculated the prediction accuracy. According to Table 1, our method significantly improved the accuracy by 2.9% over the baseline method.

#### 4.3.1. Sliding Window Cropping Module

The module crops the coarse-grained features with a sliding window strategy, which ensures that important information can be completely preserved in the subfeature blocks. For the experimental setting, we first used a 256×7×7→64 FC layer to adjust all sub-block dimensions and concatenate them to obtain a one-dimensional feature, n×64 in size. Then, we predicted the result through an FC layer. Table 1 shows that our method improved the results by 1.79% over the baseline. This shows that the proposed cropping strategy enhances the ability to acquire local features. Moreover, the fusion of local and global features greatly increases the accuracy of recognition.

Due to the different scales of cropping methods, local features with different levels of completeness will be preserved. As shown in Table 2, we investigated the impacts of multiple scale sizes on the results. We did not consider the case of stride=1 in order to balance the accuracy, running time, and computing power. The results show that when using the parameters scale=7 and stride=2, the accuracy is the highest, which indicates that the scale contains all of the important local features in the image to the greatest extent. When the scale is increased, local features contain more useless information. When the scale is reduced, the window cannot guarantee the integrity of the local features.

#### 4.3.2. Local Feature Enhancement Module

This module learns deep feature information from different receptive fields through convolutions of different scales. Local features are easily affected by noise, because in some subfeature blocks, noise may occupy most of the area, causing the model to learn features related to noise. We used enhancement loss to suppress the model’s learning of noise. We calculated the loss for each subfeature block after MBE enhancement and took the average of the loss of all sub-blocks as the loss value of the entire image. According to Table 1, this helped us to improve the accuracy by 0.45%. MBE can help the model to successfully extract more fine-grained feature information.

#### 4.3.3. Adaptive Feature Selection Module

Since our subfeature blocks do not have any prior knowledge and are random, the importance of the contribution of each sub-block to the result is also not the same. Directly fusing all sub-blocks will result in the common use of useful and useless information. The AFS module generates corresponding weights for all subfeature blocks according to their importance. Important local features will be amplified, while unimportant features will be weakened so that the model can adaptively select features that are beneficial to the prediction results. According to Table 1, our AFS module again improves the accuracy by 0.66%.

#### 4.3.4. Fusion Strategy

For the feature-level fusion strategy, we adjusted the global feature’s dimensions to obtain a 512 one-dimensional feature. We adjusted each local feature’s dimensions to 64 one-dimensional features and then concatenated them to obtain a one-dimensional feature with a size of 512 by a linear layer. According to the weight, element-wise addition was performed on the global and local features, and a linear layer was used to predict the result. For the decision-level fusion strategy, we directly predicted the expression class using the global feature and then utilized the concatenated local subfeatures, which had a size of n×64, to predict the result. Finally, the prediction results were fused according to the weight. In two sets of experiments, the weights of global and local fusion were both set to 0.5. As shown in Table 3, global features and local features are not complementary. The feature-level fusion strategy may downplay the feature information emphasized by global and local features. The decision-level fusion strategy can guarantee the independence of the respective features, and it is more reasonable to fuse only the predicted results.

#### 4.3.5. The α & β Hyperparameters

In the experiment, we used the α hyperparameter to divide the global and local proportions. The results are shown in Table 4. When α=0.5, the accuracy was the highest, which shows that the local features extracted can help the global features to supplement some fine-grained features and some detailed features that are discarded under the global receptive field.

We used the β hyperparameter as the coefficient of the enhancement loss, and we performed a series of experiments to find the optimal value. Table 5 shows that when β=0.0001, the accuracy rate was the highest. This is because the value of the enhancement loss far exceeded the global loss and local loss. If β is too large, the overall loss is almost dominated by enhancement loss, ignoring the global loss and local loss; if β is too small, the enhancement loss cannot guide our net to learn intraclass semantic information.

### 4.4. Comparisons with State-of-the-Art Methods

**Figure 7:**
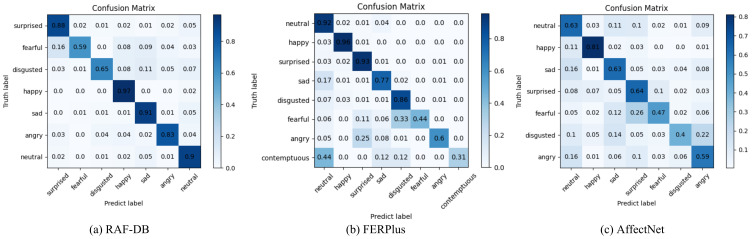
Confusion matrix of the RAF-DB, FERPlus, and AffectNet datasets. The horizontal axis represents the predicted label, and the vertical axis represents the true label. The diagonal line shows the accuracy of each type of prediction. Darker colors indicate higher accuracy.

In this section, we present comparisons with some representative methods on the RAF-DB, FERPlus, and AffectNet datasets. The confusion matrices of the three datasets are shown in Figure 7.

#### 4.4.1. Comparisons on the RAF-DB Dataset

Table 6 exhibits comparisons with existing excellent methods for the RAF-DB dataset. Our SWA-Net model exhibits the best FER result (90.03%). On the basis of also using ResNet-18 as the backbone network, our method significantly improves the prediction accuracy. Both gACNN [18] and RAN [17] use image-level cropping methods, and the former involves cropping by prior knowledge, while the latter involves mixed cropping which includes fixed size cropping and prior knowledge cropping. The accuracy levels are 85.07% and 86.90%, respectively. MA-Net [19] uses feature-level cutting and a fixed-size cutting strategy, obtaining an accuracy of 88.40%. Our method adopts a feature-level cropping and sliding window cropping strategy and is far superior to the previous three methods. Figure 7a exhibits the confusion matrix.

#### 4.4.2. Comparisons on the FERPlus Dataset

The FERPlus dataset was relabeled from the grayscale image FER2013 obtained from an Internet search and has eight types of expressions (one more “contemptuous” category than RAF-DB). Although the label noise of FERPlus is greatly alleviated compared to that of FER2013, the resolution of each image is only 48×48, which makes it a great challenge for the network to obtain information. In the experiment, we did not perform additional image enhancement operations on these images, which is consistent with RAF-DB and AffectNet. Table 7 exhibits the comparisons with the existing excellent methods on the FERPlus dataset. Our method achieved the best result with 89.22%, which shows that our SWA-Net can mine discriminant features from face images at different scales very well. Figure 7b presents the confusion matrix of the FERPlus dataset.

#### 4.4.3. Comparisons on the AffectNet Dataset

AffectNet recognition has presented a great challenge due to its complexity and diversity. We used the same 7 expressions as for RAF-DB. Table 8 exhibits comparisons with the existing excellent methods on the AffectNet dataset. Since the class distribution of AffectNet is extremely unbalanced, all existing studies included in the table adopted the oversampling strategy to expand the dataset, while we adopted the undersampling strategy, only using 12% of the original dataset to obtain the best performance. Figure 7c presents the confusion matrix.

### 4.5. Experiments on Realistic Occlusion and Pose Variation

We compared our SWA-Net with several state-of-the-art works on three occlusion datasets and six pose datasets to demonstrate its superiority for solving both facial occlusion and head pose variation problems.

According to Table 9, our SWA-Net model gave the best FER results on all three occlusion datasets. This is because our local cropping strategy helps to preserve the integrity of important features to the greatest extent, which is a problem with other cropping methods. The model further extracts fine-grained features for the coarse-grained features and can adaptively assign corresponding weights to each subfeature block. The model has the ability to select important features independently. According to Table 9, for the six pose datasets, our method still achieves the highest accuracy. Due to the various head poses, the effective area of the face is incomplete, which is similar to the occlusion situation, so it is impossible to obtain complete expression features. The difference is that different poses will cause the face in the image to change its shape, which is a problem that does not exist in occluded images. Our model focuses on compelling local features so it can autonomously find useful information from the whole image, regardless of pose changes.

In summary, in order to eliminate the facial occlusion and multiangle head pose effects associated with FER, we propose the Sliding Window Cropping strategy, which can crop out all effective local regions and guarantee the integrity of local features. Our model can adaptively focus on important regions. The Local Feature Enhancement module and Adaptive Feature Selection module lead our model to adaptively find significant regions with greater contributions to the prediction results. Therefore, our model achieves excellent results on occlusion and attitude datasets.

### 4.6. Visualizations

**Figure 8:**
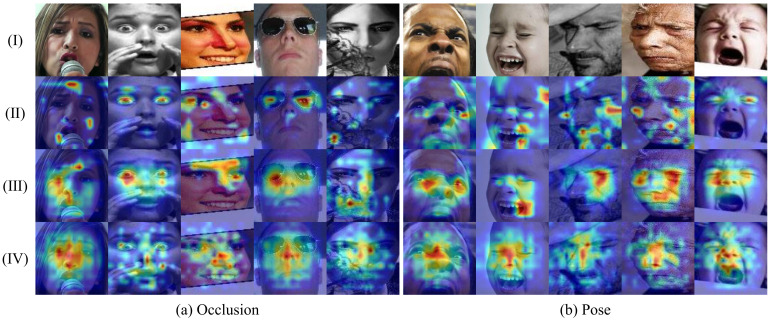
The comparisons of Grad-CAM visualization for different configurations of our method on occlusion and pose datasets. The lighter colors, the stronger Grad-CAM areas are covered. In both (**a**,**b**): (I) Raw images; (II) Base (using layer-3 of the backbone); (III) Backbone + SWC + LFE; (IV) Backbone + SWC + LFE + AFS.

To intuitively show that our method can precisely localize valid regions of facial images, we performed visualization of our proposed method through Grad-CAM [52]. We observed that, in extreme cases of facial occlusion and head pose variations, global attention is scattered, and it is difficult to concentrate on the prominent regions of the facial expression. This is because global features can only extract coarse-grained features macroscopically, and it is difficult to pay attention to multiple fine-grained feature information. This approach works well for focusing on important areas of the face in a controlled environment but is less effective in the real world, where face images are more susceptible to occlusion and poses. Our method can guide the model to mine more discriminative information from incomplete and deformed face regions caused by occlusion or poses. As shown in Figure 8, the global attention on real-world images is often scattered, and even the useful information from the image is ignored. This is especially obvious under harsh conditions, as shown in the first and fifth groups in Figure 8a, and the second and fourth groups in Figure 8b. However, our SWC and LFE modules can help the model to focus the scattered attention on the face, which is due to the excellent cropping strategy that can preserve the integrity of each local feature, and the LFE module can mine local deep-level fine-grained features, such as the first set of images in Figure 8b, where the SWC and LFE modules jointly guide the model to abandon distracting attention and focus on the eyes, nose, and mouth. In addition, in human visual cognition, the human eye can often recognize negative emotions by frowning. Therefore, in addition to paying attention to all the key regions of the face as much as possible, it is also necessary to selectively judge the importance of each region, pick out the local regions with more discriminative power in each category, and assign them higher weights. Our AFS module can first select the more important areas from the areas that the model focuses on. Just like the first group in Figure 8b, the frown is more important than the widened eyes, and it can help the model to recognize expressions more accurately, so the AFS module directs the model to shift the focus from the original eyes and noses to the frowning brows.

### 4.7. Failure Case Analysis

**Figure 9:**
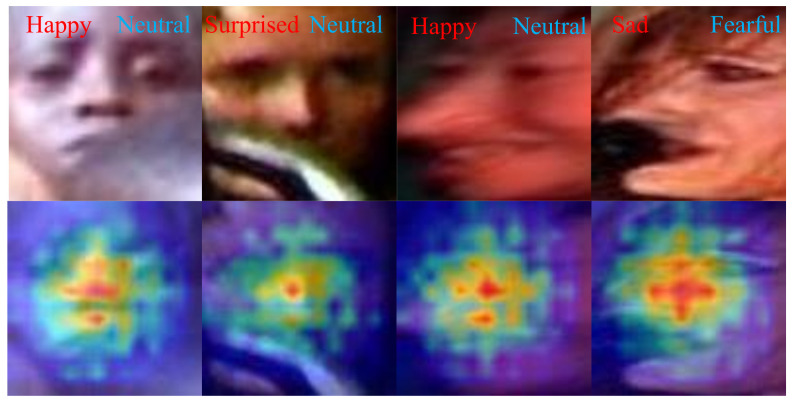
CAMs of some failure cases. The red font is the ground truth, and the blue font is the predicted label. The CAMs exhibit our model’s attention. The lighter colors, the more our model focuses on this region.

We show some failure cases in Figure 9. The proposed method cannot recognize extremely distorted faces due to the larger occlusions and pose variations present. Our SWA-Net can concentrate on the relatively important regions of faces; however, it is difficult to learn more discriminative and detailed features under such extreme conditions. Specifically, as shown in the first two columns of Figure 9, the faces are largely occluded and highly blurred, and the information with expression semantics is incomplete. Although the proposed method focused on the face regions, it failed to learn more detailed expression-related features. As shown in the last two columns of Figure 9, the face geometry of the last two cases is extremely distorted, and our method missed some key regions as the extreme deformation meant that the learned feature information was too unique and not generalizable.

## 5. Conclusions

This paper presented the SWA-Net model, which can be used to learn local discriminative and fine-grained features for FER by modeling the sliding window attention mechanism. We first proposed the Sliding Window Cropping strategy to crop local features from incomplete facial effective regions due to occlusion and poses. Then, we proposed the Local Feature Enhancement module to mine intraclass multiscale fine-grained features. We also introduced the Adaptive Feature Selection module to spontaneously address more distinguishing features from a set of local features. Extensive experiments and comparisons demonstrate that our SWA-Net model can locate and utilize the most discriminative information from occlusion or pose face images, exhibiting a high generalization ability for both facial occlusion and head pose problems. In future work, we will focus on the problem of larger facial occlusion and extremely distorted heads in FER tasks to further boost the performance of our method.

## Figures and Tables

**Table 1:** Evaluation of each module in our model on the RAF-DB dataset. ’—’ indicates that the module is not used; ’✓’ indicates that the module is used.

SWC	LFE	AFS	Acc. (%)
—	—	—	87.13
✓	—	—	88.92
✓	✓	—	89.37
✓	✓	✓	**90.03**

**Table 2:** Evaluation of the effects of different scale and stride values on the RAF-DB dataset. When using scale=7 and stride=2, the performance is the best.

	The Values of Window Parameter									
Stride	2	2	2	2	2	3	3	3	3	3
Scale	3	5	7	9	11	3	5	7	9	11
Acc. (%)	88.85	89.42	**90.03**	88.95	88.69	89.55	88.68	89.18	89.37	88.53

**Table 3:** Evaluation of Fusion Strategies on the RAF-DB dataset.

Fusion Strategy	Acc. (%)
Feature-Level Fusion	89.12
Decision-Level Fusion	**90.03**

**Table 4:** Evaluation of different α values on the RAF-DB dataset. When setting α=0.5, the performance is the best.

	The Values of α								
α	0.1	0.2	0.3	0.4	0.5	0.6	0.7	0.8	0.9
Acc. (%)	89.28	89.61	89.71	89.80	**90.03**	89.57	89.15	88.98	88.95

**Table 5:** Evaluation of different β values on the RAF-DB dataset. When setting β=0.0001, the performance is the best.

	The Values of β						
β	0.1	0.01	0.001	0.0001	0.00001	0.000001	0.0000001
Acc. (%)	86.41	86.51	87.56	**90.03**	89.57	89.31	89.24

**Table 6:** Comparisons with the state-of-the-art methods on the RAF-DB dataset.

Methods	Backbone	Year	Acc. (%)
IPA2LT [39]	ResNet (80layers)	2018	86.77
gACNN [18]	ResNet-18	2019	85.07
RAN [17]	ResNet-18	2019	86.90
SCN [40]	ResNet-18	2020	87.03
MA-Net [19]	ResNet-18	2021	88.40
DMUE [41]	ResNet-18	2021	88.76
VTFF [42]	ResNet-18	2021	88.14
RUL [27]	ResNet-18	2021	88.98
Face2Exp [43]	ResNet-50	2022	88.54
EEE [44]	VGG-Face [45]	2022	87.10
IPD-FER [30]	ResNet-18	2022	88.89
THIN [46]	VGG-16	2022	87.81
**SWA-Net (Ours)**	ResNet-18	—	**90.03**

**Table 7:** Comparisons with the state-of-the-art methods on the FERPlus dataset.

Methods	Backbone	Year	Acc. (%)
PLD [37]	VGG-13	2016	85.10
RAN [17]	ResNet-18	2019	88.55
SCN [40]	ResNet-18	2020	88.01
LDR [47]	ResNet-18	2020	87.60
RUL [27]	ResNet-18	2021	88.75
DMUE [41]	ResNet-18	2021	88.61
VTFF [42]	ResNet-18	2021	88.81
IPD-FER [30]	ResNet-18	2022	88.42
**SWA-Net (Ours)**	ResNet-18	—	**89.22**

**Table 8:** Comparisons with the state-of-the-art methods on the AffectNet dataset. Since the AffectNet dataset is extremely unbalanced, we used an undersampling strategy.

Methods	Backbone	Year	Acc. (%)
gACNN [18]	ResNet-18	2019	58.78
RAN [17]	ResNet-18	2019	59.50
DDA Loss [48]	ResNet-18	2020	62.34
IDFL [49]	ResNet-50	2021	59.20
T21DST [50]	ResNet-18	2021	60.12
SDW [51]	ResNet-18	2021	61.11
DMUE [41]	ResNet-18	2021	62.84
DMUE [41]	Res-50IBN	2021	63.11
EEE [44]	VGG-Face	2022	62.10
IPD-FER [30]	ResNet-18	2022	62.23
**SWA-Net (Ours)**	ResNet-18	—	**63.97**

**Table 9:** Comparisons with the state-of-the-art methods on the Occlusion and Pose datasets.

Datasets	Methods	Year	Occlusion	Pose ≥ 30°	Pose ≥ 45°
RAF-DB	RAN	2019	82.72	86.74	85.20
	MA-Net	2021	83.65	89.66	87.99
	**SWA-Net**	—	**87.62**	**89.71**	**89.61**
FERPlus	RAN	2019	83.63	82.23	80.40
	MA-Net	2021	—	—	—
	**SWA-Net**	—	**85.79**	**88.80**	**87.05**
AffectNet	RAN	2019	58.50	53.90	53.19
	MA-Net	2021	59.59	57.51	57.78
	**SWA-Net**	—	**60.81**	**59.92**	**61.19**

## Data Availability

Not applicable.
